# Microstructure Evolution and Mechanical Properties of High-Temperature Carburized 18Cr2Ni4WA Steel

**DOI:** 10.3390/ma17194820

**Published:** 2024-09-30

**Authors:** Zhenyang Zhang, Zehua Wu, Yuedong Yuan, Xiaonan Wang, Yanzhong Tian

**Affiliations:** 1 Key Laboratory for Anisotropy and Texture of Materials (Ministry of Education), School of Materials Science and Engineering, Northeastern University, Shenyang 110819, China; 2200722@stu.neu.edu.cn; 2 School of Iron and Steel, Soochow University, Suzhou 215137, China; 20224049005@stu.suda.edu.cn; 3 Changshu Tiandi Coal Machinery Equipment Co., Ltd., Suzhou 215500, China; csyyd@126.com

**Keywords:** high-temperature carburization, 18Cr2Ni4WA steel, mechanical property, carbon concentration, carbide

## Abstract

Surface carburized steels are extensively utilized in gears due to their exceptional properties. The quality of the carburized layer is crucial in enhancing the contact fatigue and wear resistance of the components. However, the conventional carburizing method takes a long time and induces a carbon emissions problem. In this study, the 18Cr2Ni4WA steel was double tempered (650 °C/4 h) after carburizing at 930 °C and 950 °C. The microstructural evolution, carbide precipitation, and mechanical properties of different carburized layers were analyzed. The results showed that increasing the carburizing temperature can control the microstructure of the carburized layer while reducing the carburizing time by over 60%. The high carbon content improves the strength of the carburized materials at 950 °C, and the inhibition of dislocation motion and grain boundary by the precipitation of more carbides ensures the stability of grain size, maintaining the strength of the materials. The carburized specimens at 950 °C showed an excellent combination of strength and plasticity in different carburized layers due to the variations in solid solution strengthening, dislocation strengthening, precipitation strengthening, and grain boundary strengthening induced by carbon atoms. This study holds significant reference for the advancement of modern steels carburized at high temperatures in a short time.

## 1. Introduction

Heavy-duty gears experience significant stresses during operation. The root part of the gear undergoes bending stresses, while the gear surface experiences rolling and sliding contact stresses. Accordingly, the gear material must exhibit high strength in the surface layer and high toughness in the core. Researchers have introduced a gradient microstructure on the surface through elemental penetration (such as carburization, nitrogen, boron, etc.), enhancing the surface strength and abrasion resistance while maintaining good toughness inside the gears. This increases the service life of the gears and ensures their safety performance [1,2,3]. The conventional carburizing process has many disadvantages, such as lengthy cycle times, poor efficiency, and high carbon emissions [4,5]. In order to improve the performance of gear steels, researchers have explored strategies for fine grain strengthening, solid solution strengthening, and precipitation strengthening. However, substantial costs and complex procedures are desired to achieve these enhancements, which often come with substantial costs and complex procedures [6,7].

Notably, 18Cr2Ni4WA steel has a low carbon content that is highly hardenable and relatively inexpensive. The application of the carburizing process results in the formation of a high-strength surface layer and a high-toughness core. The superior properties of this steel render it an optimal material for fabricating components, such as heavy-duty gears [8,9]. The surface carbon content can be increased by carburizing, thus demonstrating its hardness and mechanical properties [10,11]. In parallel, it helps the surface withstand mechanical wear because of the significant correlation between hardness and wear characteristics [12]. Coarse plate martensite and high austenite content tend to form in the 18Cr2Ni4WA steel, reducing surface strength and deteriorating service performance [13,14,15]. To reduce the retained austenite content and improve surface strength, high-temperature tempering and secondary quenching were widely used [10,16,17]. Pan et al. [18] found that carbides can significantly increase surface hardness at carbon contents up to 0.5 wt%. The carbon atoms precipitated as carbides and aggregated around the dislocation and granular boundaries, leading to a reduced width of the martensite lamellae. Therefore, the material can achieve high strength and toughness after tempering [19,20,21]. Additionally, high-temperature tempering can remove retained austenite and improve the toughness. However, it has been a challenge to further improve the overall properties of the carburized layer by heat treatment [22,23,24].

It is found that high carburizing temperatures can accelerate carbon diffusion, reduce the carburizing time, increase the carbon content of the hardened layer, and improve the surface properties [25]. Most studies were devoted to evaluating the mechanical properties of 18Cr2Ni4WA at conventional carburizing temperatures [24,26]. In contrast, fewer studies were conducted on the high-temperature (>930 °C) carburizing strategy [27]. It is important to note that raising the carburizing temperature can affect the grain size, the martensitic phase, carbide transformation, retained austenite, as well as the interplay of these complex microstructures after carburizing and quenching, which collectively contribute to the mechanical properties of the steel [28,29,30]. Therefore, it is essential to investigate the hardness, tensile properties, and microstructure changes of gear steel layer by layer.

In this work, we proposed a high-temperature carburizing process that can achieve a carburized depth of about 5 mm within a relatively short time. Two high-temperature tempering processes were adopted to significantly reduce the retained austenite content of the carburized layer in order to facilitate machining as well as to improve the performance of subsequent secondary quenching [31]. In addition, the microstructures and mechanical properties of different carburized layers were analyzed, and the main strengthening modes of the carburized layers were quantified, which are important for the development of an efficient carburizing process.

## 2. Materials and Methods

**Figure 1:**
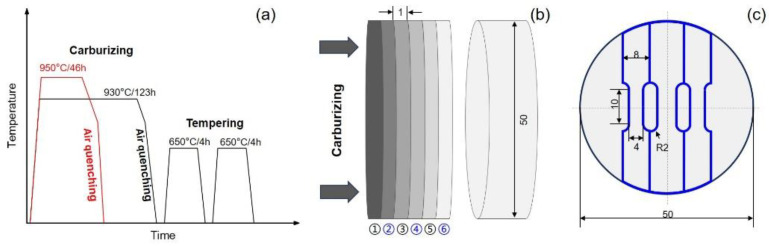
Schematic illustration on the carburizing process and sample preparation. (**a**) Different carburizing processes and heat treatments; (**b**) layer-by-layer samples of carburized steel; (**c**) geometry of tensile specimen (unit: mm).

The chemical composition (in wt%) of the 18Cr2Ni4WA steel is Fe-0.14C-4.14Ni-1.45Cr-0.79W-0.46Mn-0.24Si. The Φ50 mm cylinder was treated in a gas carburizing furnace. Figure 1a shows the carburizing and heat treatment procedures. The samples were gas-carburized using a pit-type carburizing furnace (RQD-90–9). The carburizing agents used were nitrogen and methanol. Two carburization processes of 123 h at 930 °C and 46 h at 950 °C were compared. The carbon potentials were 1.15 wt% and 1.25 wt% for the heavy carburizing period and 0.82 wt% and 0.85 wt% for the diffusion period, and the ratios of heavy carburizing time to diffusion were 17:3 and 3:1, respectively. The carburized samples were cooled to 820 °C in the furnace and then cooled in air. High-temperature tempering was conducted at 650 °C. The carburized specimens at 930 °C and 950 °C were referred to as 930CT and 950CT, respectively. Slices with a thickness of 1 mm were cut layer by layer along the carburizing direction, as shown in Figure 1b. Figure 1c showed the tensile specimens from the 2nd, 4th, and 6th slices, and the corresponding carburization depths were defined as 1.5 mm, 3.5 mm, and 5.5 mm.

The hardness was measured using an MH-500 Vickers (Future-Tech, Kawasaki, Japan) hardness tester with a load of 500 g. Tensile tests were performed at room temperature using an AG-XPLUS (Shimadzu, Beijing, China) universal testing machine at a strain rate of 10^−3^ s^−1^, and the strain was measured with a SANJING-Y10/10 mechanical extensometer until fracture. Samples of 10 × 10 × 1 mm were cut from the carburized layer, and the surfaces were polished and examined by X-ray diffraction (XRD) at a scanning speed of 2°/min and a scanning range of 40–100°. Scanning electron microscopy (Zeiss Auriga Compact FIB-SEM, Oberkochen, Germany) and electron back scatter diffraction (EBSD) were used for microstructural analysis. Prior to testing, the samples were mechanically polished and etched with a 4 vol% alcoholic solution of nitric acid. The EBSD characterization was conducted at an accelerating voltage of 20 kV and a step size of 100 nm. The area fraction and average diameter of undissolved carbides were measured using Image-Pro Plus 6.0 software and the binarization method.

## 3. Results

### 3.1. Carbon Content and Mechanical Properties

**Figure 2:**
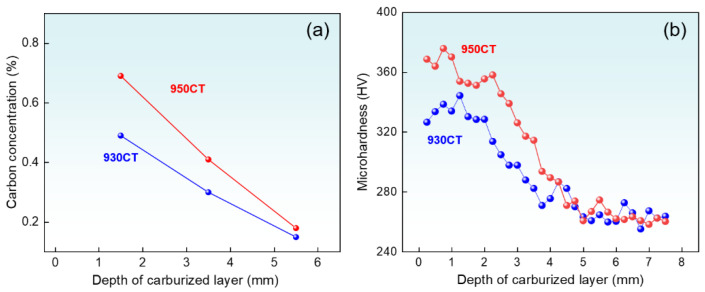
Plots of the (**a**) carbon concentration and (**b**) microhardness against the carburized depth.

Figure 2 shows the carbon content and hardness distributions in the carburized layer of the two samples. The carbon content of 950CT is higher than 930CT in the same depth. The difference between the carbon content of the two samples gradually decreases as the depth of the carburized layer increases. The variation of hardness with carburization depth is similar to the carbon content, as shown in Figure 2b. The hardness at the carburized depth of 5 mm remains basically the same for the two specimens, achieving a carburization depth of about 5 mm. It should be noted that the hardness of the near-surface layer (1 mm) is higher than that of the surface layer (0.2 mm), which is due to the decarburization of the surface during heat treatment [32].

**Figure 3:**
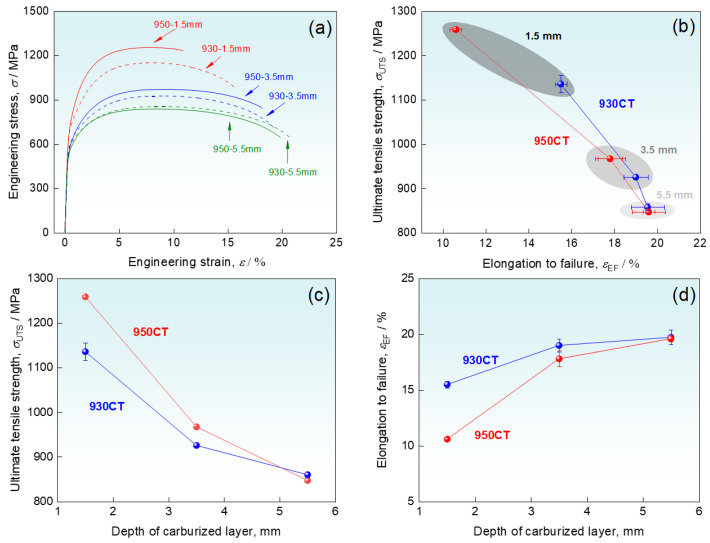
Mechanical properties of specimens in different carburized depths (**a**) Engineering stress-strain curves; (**b**) ultimate tensile strength and elongation to failure of the specimens in different carburized depths; and (**c**) ultimate tensile strength and (**d**) elongation to failure against the carburized depth.

Figure 3a shows the engineering stress-strain curves at carburized depths of 1.5 mm, 3.5 mm, and 5.5 mm. It can be seen that the yield strength and tensile strength of the carburized layer increased after high-temperature carburizing treatment. With the increase of the carburized depth, the tensile strength decreased and the elongation increased for both carburized specimens, as shown in Figure 3b. The tensile strength of the 950CT reaches 1260 MPa at the depth of 1.5 mm, which is 10% higher than the 1136 MPa of the 930CT. The tensile strength of both specimens decreases significantly at the depth of 3.5 mm, where the tensile strength of 930CT decreases to 926 MPa and 950CT decreases to 967 MPa. At the depth of 5.5 mm, the tensile strengths are comparable for the two kinds of specimens, as shown in Figure 3c. Figure 3d shows the elongation plotted against the carburized depth. The elongation of specimen 950CT is much lower than that of 930CT at the depth of 1.5 mm, and the difference becomes insignificant with increasing the depth.

### 3.2. Microstructures in Different Carburized Layers

**Figure 4:**
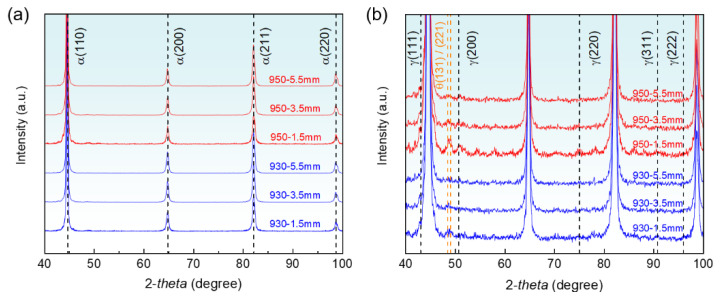
XRD spectra of specimens with different carburizing temperatures at 1.5 mm, 3.5 mm, and 5.5 mm. (**a**) Complete and (**b**) localized diffraction peaks.

Figure 4 shows the XRD spectra of tensile specimens at different carburized depths (1.5 mm, 3.5 mm, and 5.5 mm). The material consists of tempered martensite (α-ferrite and θ-carbide) and a small amount of retained austenite (γ). Figure 4a shows the highest peaks of α (110), (200), (211), and (220) at adepth of 1.5 mm. As the carburizing temperature increases, Figure 4b shows the integral area of θ-carbide and γ-retained austenite diffraction peaks increased. The θ (131), (221), and γ (200) peaks gradually disappear towards the core of the carburized layer, while the α (110), (200), (211), and (220) peaks are enhanced. The difference in carbon content variation decreases towards the core of the carburized layer, resulting in the convergence of the α diffraction peaks at the depth of 5.5 mm.

**Figure 5:**
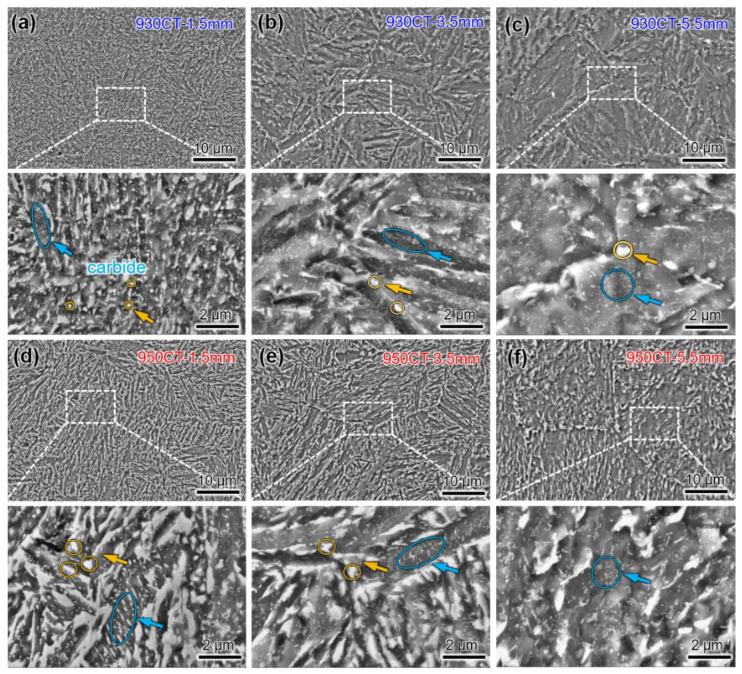
SEM images of specimens (**a**–**c**) 930CT and (**d**–**f**) 950CT at carburized depths of (**a**,**d**) 1.5 mm, (**b**,**e**) 3.5 mm and (**c**,**f**) 5.5 mm.

Figure 5 shows the microstructure at different carburized depths. At a carburized depth of 1.5 mm, profuse fine carbides are indicated by the blue arrows distributed inside the martensite. The volume fraction rises with the increase of carburizing temperature, and the morphology changes from spherical shape to short rod shape. The yellow arrows indicate the undissolved carbide that is enriched with Cr and W elements, which can play a role in pinning grain boundaries, as shown in Figure 5a,d [33]. The number of grain boundaries decreases with increasing depth, which is due to the reduction of undissolved carbides at the grain boundaries, as shown in Figure 5b,e. The carbide content of the specimen is stable at the depth of 5.5 mm, and the cementite size is finer and unaffected by the carburizing temperature, as shown in Figure 5c,f.

### 3.3. Grain Size at Different Carburizing Temperatures

**Figure 6:**
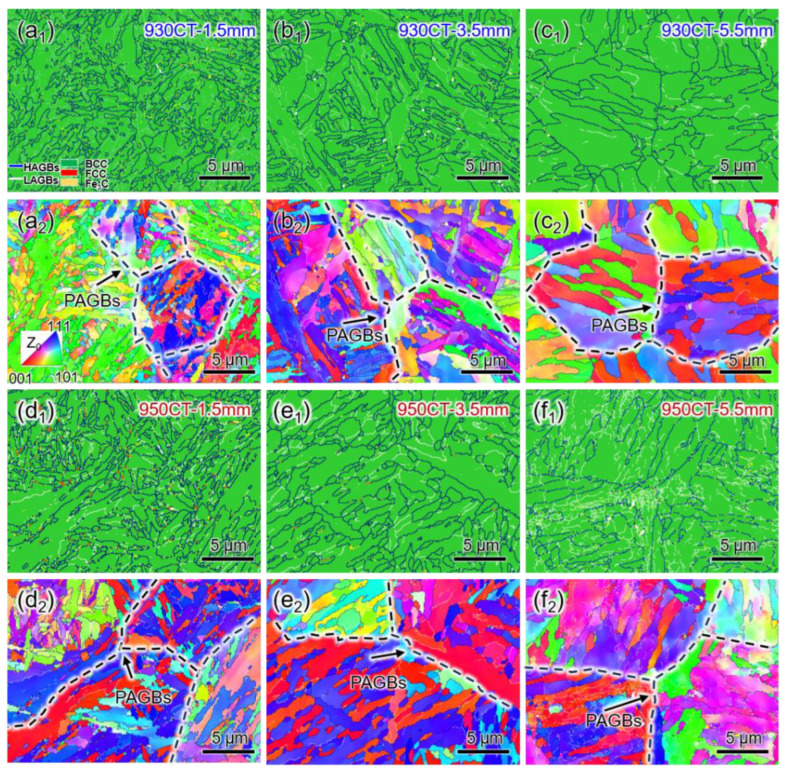
(**a_1_**–**f_1_**) Phase maps and (**a_2_**–**f_2_**) IPFs of specimens (**a**–**c**) 930CT and (**d**–**f**) 950CT at carburized depths of (**a**,**d**) 1.5 mm, (**b**,**e**) 3.5 mm, and (**c**,**f**) 5.5 mm.

Figure 6 shows the microstructures at different carburized depths. The prior austenite grains of 950CT specimens are coarser than the 930CT specimens, and prior austenite grain boundaries (PAGBs) can be observed from the inverse pole figures (IPFs). The mobility of supersaturated carbon atoms was enhanced in the martensite during the high-temperature tempering process, allowing the migration and eventually transforming the quenched martensite into ferrite [24].

Microstructures at different carburized depths were described in the following. The sample at the carburized depth of 1.5 mm has the highest solid-solution carbon content and the greatest degree of lattice distortion, retaining apparent martensite microstructure. It is confirmed that these carbides were Fe3C with minimal residual austenite, which precipitated around grain boundaries and limited the expansion of ferrite or martensite grain size when the carburizing temperature is increased. The grain size is represented by the equivalent grain area in EBSD due to the complexity of the microstructure, which increased from 2.5 μm to 2.8 μm with increasing carburizing temperature, as shown in Figure 6a,d. Figure 6b,e shows that the specimens exhibit a significant decrease in carbon content at the depth of 3.5 mm, which mitigated the matrix lattice distortion. Additionally, the low carbon content reduces the carbide content, weakening the pinning effect on grain boundaries and coarsening the grain size. Figure 6c,f shows that the microstructures of both samples continue to transform from martensite to ferrite at a depth of 5.5 mm, resulting in coarsened grain sizes of 5.9 μm and 6.6 μm, respectively. The coarsened grain size and decreased carbon content result in a continuous decrease in strength with depth.

## 4. Discussion

### 4.1. Effect of Carbon Content on Microstructures in Different Carburized Layers

It was found that high carburizing temperature favors carbon diffusion, which results in the high carbon content of the carburized layer. The γ phase lattice is hard to shear due to the high carbon concentration. The initially formed martensite inhibits the formation of additional martensite, leading to a small and densely distributed martensite structure during the cooling process [19]. However, the enhanced carbon solid solubility also increases the stability of austenite, resulting in a number of retained austenites after cooling, which reduces the strength and increases the tendency to crack. In this work, the elimination of retained austenite by high-temperature tempering reduced the strength of the material, thus minimizing the complex effect of retained austenite on strength. In parallel, the carbon atoms underwent long-range diffusion and precipitated as carbides, which alleviated the lattice distortion. The martensite gradually transformed into ferrite, making it possible to quantify the mechanical properties after high-temperature carburizing treatment [16,34].

Figure 4b shows that the diffraction peaks of retained austenite are quite weak, indicating that the effect of carburizing temperature on its content is low, thus it can be assumed that it contributed negligibly to the strength [35,36,37]. The strength of martensitic steels is affected by following main factors [19]: (1) solid solution strengthening $\sigma_{SS}$, (2) dislocation strengthening $\sigma_{DS}$, (3) precipitation strengthening $\sigma_{P}$, and (4) grain boundary strengthening $\sigma_{GB}$. Based on the strong correlation between strength and hardness [38,39], researchers calculated the strengthening contribution [40]:

(1) $${H_{v}=0.4(\sigma }_{SS}+\sqrt{{(\sigma }_{DS}{+\sigma }_{GB}{)}^{2}+{{\;\sigma }_{P}}^{2}}+110)$$

At the carburized depth of 1.5 mm, the high carbon concentration results in a pronounced martensitic structure retained by a greater degree of lattice distortion. Figure 6a,d shows that most of the carbides precipitate at the martensite boundaries and dislocations, leading to the simultaneous enhancement of $\sigma_{SS}$ and $\sigma_{DS}$ [18]. The strength increment from solid solution strengthening is calculated as [41]:

(2) $$\sigma_{SS}=\sum_{i}(\beta_{i}^{2}x_{i}{)}^{1/2}$$

where $\beta_{i}$ is the solid solution constant of Fe-based alloys and $x_{i}$ is the atom fraction of the alloying element. The strength of dislocation strengthening $\sigma_{DS}$ is calculated as [42]:

(3) $$\sigma_{DS}=\alpha MbG\sqrt{\rho }\;$$

(4) $$\rho =14.4\frac{\varepsilon^{2}}{b^{2}}$$

where α = 0.3 is a coefficient [43], M = 3 is the Taylor orientation factor, G = 64 GPa is the shear modulus, b = 0.248 nm is the Burgers vector, ε is the lattice strain produced by carbon redistribution, which is calculated from the diffraction peak broadening [28]:

(5) $$\beta_{(hkl)L}\left(2\theta \right)+\beta_{(hkl)\varepsilon }\left(2\theta \right)=\frac{0.9\lambda }{L}sec\theta_{hkl}+2\varepsilon \;tan\theta_{hkl}$$

where $\beta_{(hkl)L}\left(2\theta \right)$ and $\beta_{(hkl)\varepsilon }\left(2\theta \right)$ are the broadening due to lattice distortion and grain size, θ is the Bragg angle corresponding to the main diffraction peak, and λ is the wavelength of X-ray. In this work, the grain sizes are in the micron range; hence, the amount of broadening caused by microstructure refinement can be ignored [44].

The high tempering temperature increases the activity of carbon atoms to precipitate carbides in the high-carbon retained austenite, and the diffuse distribution of fine carbides hinders the dislocation slip through the non-deformable particles to further improve the matrix strength, which is calculated by the Orowan bypass mechanism as follows [45]:

(6) $$\sigma_{p}=0.26\frac{Gb}{r_{P}}f_{p}^{1/2}ln(\frac{r_{p}}{b})$$

where $f_{p}$ and $r_{p}$ are the volume fraction and average radius of precipitations, respectively. Therefore, at the depth of 3.5 mm, the two specimens are aggravated by grain size and precipitated phase radius and distribution.

The carbon content of both specimens at the depth of 5.5 mm is further decreased compared to that at 3.5 mm. During the tempering process, the carbon atoms are sufficiently dissolved, resulting in the microstructure characterized by equiaxed ferrite morphology, along with a reduction in both the number and size of carbides. As the martensite volume fraction decreases, the grain size becomes the dominant factor in strength. The grain boundary strength ($\sigma_{GB}$) can be obtained by using the Hall–Petch relationship [46]:

(7) $$\sigma_{GB}=k_{Y}/\sqrt{D}$$

where D is the effective grain size, with $k_{Y}$ = 0.62 MN m^−1.5^ as the Hall–Petch coefficient. Therefore, the strengthening mechanism at the depth of 5.5 mm for both specimens is dominated by grain boundary strengthening. The different microstructure parameters of martensitic steels are summarized in Table 1.

### 4.2. Effect of Carburizing Temperature on Strength at Different Locations

High carburizing temperatures can increase the carbon content of the carburized layer and the prior austenite grain size. According to the discussion in Section 4.1, the carbon content seriously affects the martensite strengthening mechanism, which in turn leads to different microstructures and properties of each layer.

At the depth of 1.5 mm, the carbon content of 950CT is approximately 0.7%, while that of 930CT is approximately 0.5%. The increase in solid solution carbon concentration produces significant lattice distortion, which increases the dislocation density after martensitic phase transformation. After tempering, a large number of carbides precipitated, which increased the deformation resistance of the martensite. According to Table 1, the strengthening effect of high-carbon specimens originates mainly from the lattice distortion caused by solid-solution carbon atoms and the defect density introduced by the martensitic phase transformation. The increased volume of precipitates improves the precipitation-strengthening effect of high-temperature carburization. The carbides enriched at the grain boundaries play a pinning role in the grain boundary migration, restricting the excessive growth of martensite grain size. However, it plays a trace effect on the strength of the specimen. In summary, the increased $\sigma_{SS}$ and $\sigma_{DS}$ make the strength of 950CT higher than that of 930CT.

At the depth of 3.5 mm, the carbon content decreases to 0.41% (950CT) and 0.3% (930CT) compared to a depth of 1.5 mm, releasing the lattice distortion and decreasing the defect density after the phase transition, which ultimately leads to a reduction in the $\sigma_{SS}$ and $\sigma_{DS}$. In parallel, the coarse martensite size leads to a reduction in the $\sigma_{GB}$ but the finer carbides lead to an increase in the $\sigma_{P}$ than the depth of 1.5 mm. In this case, there is no significant difference in the average radius of precipitates between 950CT and 930CT, whereas the higher temperature carburization results in coarser grain sizes in the 950CT specimens. In parallel, a higher volume fraction of precipitates is obtained, resulting in improved strength.

At the depth of 5.5 mm, the carbon contents of the two specimens are 0.17% (950CT) and 0.15% (930CT), which are quite similar. In this case, the lattice distortion caused by solid solution atoms is weakened, and the solid solution strengthening and dislocation strengthening decrease. Meanwhile, the volume fraction and average radius of carbides are at the same level, leading to the convergence of the precipitation strengthening effect. The high carburizing temperature leads to coarse prior austenite and ferrite grain sizes in the 950CT sample, which ultimately leads to a reduction in strength. Based on the microstructural parameters of the 930CT and 950CT samples in Table 1, the contributions of each strengthening mechanism were quantitatively assessed in Table 2. It should be noted that the precipitated carbon atoms decreased the actual solid solution strengthening, thus increasing the calculated HV1 by Equation (1) over the measured HV0.

## 5. Conclusions

In this work, the 18Cr2Ni4WA gear steel was treated using carburization at 930 °C and 950 °C and then twice tempering at 650 °C. High-temperature carburizing at 950 °C increases carburizing efficiency by more than 60% of conventional carburizing at 930 °C. This process not only contributes to advances in the field of materials engineering and manufacturing but also supports the sustainable development of the industry. The microstructure and mechanical properties at the carburized depths of 1.5 mm, 3.5 mm, and 5.5 mm are elucidated. The main conclusions are summarized as follows:

1. The 950 °C carburized sample achieved a higher level of carbon content, resulting in a high carburized layer of 5 mm. High carbon content and high-density dislocations are retained at the depth of 1.5 mm, and the grain boundaries are pinned efficiently. The strength is enhanced by a higher degree of solid solution strengthening and dislocation strengthening, which make the 950 °C carburized sample excellent comprehensive performance without excessive loss of plasticity.
2. The difference in carbon content between the two samples is not significant, and the pinning effect of carbides on the boundaries is weakened at the depth of 3.5 mm. High-temperature carburizing increases the grain size and the density of carbides, leading to a decrease in grain boundary strengthening and an increase in precipitation strengthening. The bilateral effects make the slightly increased strength for the 950 °C carburized sample.
3. At a depth of 5.5 mm, the carbon contents are similar for the two specimens and greatly reduced. Equiaxed ferrite is obtained after tempering, and the grain size becomes the dominant factor for strengthening, which ultimately leads to the strength decrease after high-temperature carburizing.

## Figures and Tables

**Table 1:** Summary of the microstructure parameters of the 930CT and 950CT samples.

Samples	ρ/10^14^ m^−2^	D/μm	f_p_/%	r_P_/μm
930CT-1.5mm	2.782	2.5	7.59	0.049
950CT-1.5mm	4.079	2.8	12.68	0.069
930CT-3.5mm	1.652	3.3	6.37	0.034
950CT-3.5mm	1.114	4.9	9.37	0.036
930CT-5.5mm	0.293	5.9	4.69	0.027
950CT-5.5mm	0.244	6.6	5.64	0.031

**Table 2:** Summary of calculated strengthening contribution from each mechanism in the 930CT and 950CT samples.

Samples	$\sigma_{SS}$/MPa	$\sigma_{D}$/MPa	$\sigma_{GB}$/MPa	$\sigma_{P}$	HV1	HV0
930CT-1.5 mm	365.6	268.1	392.1	121.1	458.7	334.1
950CT-1.5 mm	470.1	324.6	370.5	119.9	514.1	370.2
930CT-3.5 mm	288.2	169.7	343.4	150.8	373.2	297.9
950CT-3.5 mm	366.1	206.6	281.2	174.7	397.7	326.2
930CT-5.5 mm	236.3	87.1	255.3	154.6	289.1	263.5
950CT-5.5 mm	244.7	79.4	241.3	156.3	284.3	260.8

## Data Availability

The raw data supporting the conclusions of this article will be made available by the authors on request.
